# Delivery of Human Stromal Vascular Fraction Cells on Nanofibrillar Scaffolds for Treatment of Peripheral Arterial Disease

**DOI:** 10.3389/fbioe.2020.00689

**Published:** 2020-07-17

**Authors:** Caroline Hu, Tatiana S. Zaitseva, Cynthia Alcazar, Peter Tabada, Steve Sawamura, Guang Yang, Mimi R. Borrelli, Derrick C. Wan, Dung H. Nguyen, Michael V. Paukshto, Ngan F. Huang

**Affiliations:** ^1^Veterans Affairs Palo Alto Health Care System, Palo Alto, CA, United States; ^2^Fibralign Corporation, Inc., Union City, CA, United States; ^3^The Stanford Cardiovascular Institute, Stanford University, Palo Alto, CA, United States; ^4^Department of Cardiothoracic Surgery, Stanford University, Palo Alto, CA, United States; ^5^Division of Plastic and Reconstructive Surgery, Stanford University, Palo Alto, CA, United States

**Keywords:** angiogenesis, peripheral arterial disease, stem cell therapy, aligned scaffold, anisotropy, hindlimb ischemia

## Abstract

Cell therapy for treatment of peripheral arterial disease (PAD) is a promising approach but is limited by poor cell survival when cells are delivered using saline. The objective of this study was to examine the feasibility of aligned nanofibrillar scaffolds as a vehicle for the delivery of human stromal vascular fraction (SVF), and then to assess the efficacy of the cell-seeded scaffolds in a murine model of PAD. Flow cytometric analysis was performed to characterize the phenotype of SVF cells from freshly isolated lipoaspirate, as well as after attachment onto aligned nanofibrillar scaffolds. Flow cytometry results demonstrated that the SVF consisted of 33.1 ± 9.6% CD45^+^ cells, a small fraction of CD45^–^/CD31^+^ (4.5 ± 3.1%) and 45.4 ± 20.0% of CD45^–^/CD31^–^/CD34^+^ cells. Although the subpopulations of SVF did not change significantly after attachment to the aligned nanofibrillar scaffolds, protein secretion of vascular endothelial growth factor (VEGF) significantly increased by six-fold, compared to SVF cultured in suspension. Importantly, when SVF-seeded scaffolds were transplanted into immunodeficient mice with induced hindlimb ischemia, the cell-seeded scaffolds induced a significant higher mean perfusion ratio after 14 days, compared to cells delivered using saline. Together, these results show that aligned nanofibrillar scaffolds promoted cellular attachment, enhanced the secretion of VEGF from attached SVF cells, and their implantation with attached SVF cells stimulated blood perfusion recovery. These findings have important therapeutic implications for the treatment of PAD using SVF.

## Introduction

Peripheral arterial disease (PAD) affects over 10 million people in the United States (Benjamin et al., 2018). It is associated with reduced blood flow to the arms and legs, leading to pain and even limb amputation. Major risk factors include smoking, diabetes mellitus, and aging. Severe cases of PAD lead to critical limb ischemia (CLI) that is characterized by rest pain, gangrene formation, and possible amputation of the limb (Davies, 2012). Current clinical treatments of PAD involve surgical interventions including bypass grafting and angioplasty to restore blood flow to the affected limb. However, a large portion of patients with severe disease lack suitable vessels for vascular intervention (Gerhard et al., 1995). Therefore, there is an urgent need for alternative approaches to stimulate angiogenesis.

Cell-based therapies hold promise for regenerating neovessels that support blood flow in the ischemic limb. Stem cells including bone marrow-derived mesenchymal stem cells (MSCs) are a potential therapy as they are easy to expand, resilient in hypoxic conditions, and secrete paracrine factors that have angiogenic effects (Kinnaird et al., 2004). Although some clinical studies report that the delivery of MSCs to the site of limb ischemia significantly improved rest pain and increased the ankle brachial index as a measurement of vascular function, limb amputation rates did not significantly change with treatment (Lu et al., 2011; Gupta et al., 2013). Similarly, clinical trials using mononuclear cells from bone marrow or peripheral blood have also showed mixed results (Rigato et al., 2017; Qadura et al., 2018). Consequently, alternative therapeutic cell types are desired for treatment of PAD.

The stromal vascular fraction (SVF) is a heterogeneous population of cells that is derived from subcutaneous fat (Levi et al., 2011; Blackshear et al., 2017). SVF contains various types of cells including adipose stromal and hematopoietic stem and progenitor cells, endothelial cells, erythrocytes, fibroblasts, lymphocytes, monocyte/macrophages, and pericytes (Zuk et al., 2001, 2002; Bourin et al., 2013). Cells from the SVF release a mix of cytokines and therapeutic growth factors such as vascular endothelial growth factor (VEGF) that promote angiogenesis (Thangarajah et al., 2009; Kapur and Katz, 2013; Comella et al., 2016). Compared to other cell types that require *in vitro* expansion, SVF can be derived autologously, extracted in a minimally invasive manner in a clinical setting (Levi et al., 2011), and transplanted back within hours. Consequently, SVF may have greater translational relevance than other stem cells types for treatment of limb ischemia. We have previously shown that collagen scaffolds seeded with human SVF and subcellular populations thereof significantly improved revascularization to dermal wounds (Brett et al., 2017b), which supports the safety of SVF-seeded collagen scaffolds.

Regardless of the kind of stem cell used, a major limitation to stem cell therapy is poor survival of the cells when transplanted in saline. As an alternative to saline as a cell delivery vehicle, biological scaffolds can localize cell delivery to the site of the scaffold, while also providing important extracellular matrix cues that modulate the survival and angiogenic capacity of the transplanted cells. In particular, cues derived from nano-scale anisotropic patterns of fibrillar collagen can modulate cellular organization, growth factor secretion, and upregulation of integrin gene expression (Huang et al., 2013a, b; Nakayama et al., 2015, 2019). We have previously demonstrated that parallel-aligned nanofibrillar scaffolds promote the survival and angiogenic capacity of transplanted primary human endothelial cells or human induced pluripotent stem cell-derived endothelial cells in a mouse model of PAD (Huang et al., 2013b; Nakayama et al., 2015). These studies suggest that nanoscale spatial patterning cues can directly modulate biological functions of therapeutic cells upon transplantation into the ischemic limb. Toward clinical translation, these nanofibrillar scaffolds have been demonstrated to improve angiogenesis (Huang et al., 2013b), arteriogenesis (Nakayama et al., 2015), and lymphangiogenesis (Hadamitzky et al., 2016) *in vivo*. Recently, these aligned collagen scaffolds have been demonstrated to be safe in a clinical study, without complication at a 1 year follow up (Rochlin et al., 2020). Accordingly, the delivery of SVF using aligned nanofibrillar scaffolds may synergistically enhance revascularization for treatment of PAD.

Therefore, the objective of this study was to characterize the adherent cells from human SVF on aligned nanofibrillar scaffolds, and to assess the therapeutic potential of aligned nanofibrillar scaffolds seeded with SVF in a murine model of PAD. We show that the subpopulation of SVF cells that adhered to the nanofibrillar scaffold had significantly higher release of VEGF, but without a significant change in the CD45^–^/CD31^–^/ CD34^+^ cell fraction, compared to bulk SVF cells in suspension. When implanted into mice with induced hindlimb ischemia as an experimental model of PAD, the SVF-seeded scaffolds significantly increased blood perfusion, compared to cell delivery in saline. These findings suggest that aligned nanofibrillar scaffolds promoted the adhesion of SVF cells that induce angiogenesis and blood perfusion recovery, which has important therapeutic implications for the treatment of PAD.

## Materials and Methods

### Fabrication of Aligned Nanofibrillar Scaffolds

The aligned nanofibrillar collagen scaffolds were fabricated using shear-based fibrillogenesis technique as described previously (Huang et al., 2013b). In brief, purified monomeric type I collagen solution was concentrated to reach a liquid crystal state (Bobrov et al., 2002; Paukshto et al., 2008) and then sheared onto a rigid surface, creating thin film formed by parallel-aligned nanofibrils with 200–300 nm diameter (Muthusubramaniam et al., 2012). To make three-dimensional thread-like scaffolds, the membranes were dissociated from the rigid surface into a free-standing film that self-assembled by liquid–air surface tension into the thread-like scaffold (McMurtry et al., 2008). The scaffolds were then crosslinked by 1-ethyl-3-(3-dimethylaminopropyl)-1-carbodiimide (EDC) hydrochloride chemistry at 1 mg/ml and sterilized by e-beam per standard protocols (Fibralign Corporation). Surface topography of the scaffolds was assessed by routine scanning electron microscopy (SEM) (Huang et al., 2013b).

### Isolation of SVF

Lipoaspirate was obtained from healthy female patients (*n* = 6) undergoing elective procedures in accordance with the Stanford University Institutional Review Board and kept at 4°C until processing. All samples were processed within 24 h from the time of collection. SVF cells were isolated based on established methods (Tevlin et al., 2016). Lipoaspirate was rinsed twice with equal volume of phosphate buffered saline (PBS) to separate fat from blood. Fresh collagenase digestion buffer was prepared using M199 medium containing 2.2 mg/ml type II collagenase (Sigma–Aldrich), 1000 U/ml DNAse, 0.5 μM calcium chloride, 0.1% bovine serum albumin, 1% polaxamer-188 (Sigma–Aldrich), and 2% hydroxyethyl piperazine ethanesulfonic acid (Life Technologies), and filtered using a 0.22-μm filter system. Aliquots of the rinsed fat (12.5 ml) were transferred into 50-ml Falcon tubes, and an equal volume of collagenase digestion buffer was added to the fat. The tube caps were sealed with Parafilm (Bemis NA). The fat/collagenase mixture was incubated at 37°C in a water bath for 10 min to activate the collagenase. The tubes with fat/collagenase mixture were placed into the orbital shaker set at 220 r/min for 45 min. Collagenase activity was then neutralized by addition of an equal volume of cold buffer consisted of PBS containing 2% fetal bovine serum, 1% poloxamer-188, and 1% penicillin/streptomycin (FACS buffer). The solution was then centrifuged at 1250 r/min at 4°C for 10 min. Supernatant was aspirated and the SVF pellets were resuspended again in FACS buffer again and filtered through a 100-μm cell strainer, before centrifuging again at 1250 r/min at room temperature for 10 min. The resulting cell pellet was resuspended in 15 ml FACS buffer and carefully layered over 15 ml of room-temperature equilibrated Histopaque (Sigma–Aldrich) in a new 50-ml Falcon tube. The cell suspension with Histopaque was centrifuged at 1500 r/min for 30 min at room temperature with acceleration set to low and deceleration settings inactivated. The cloudy interface was transferred to a new 50 ml Falcon tube, and 40 ml FACS buffer was added. The cell suspension was centrifuged at 1500 r/min at 4°C for 5 min. The pellet was resuspended for cell culture or for flow cytometry analysis.

### Flow Cytometry

At indicated time points, cells were incubated for 30 min on ice in FACS buffer containing antibodies to CD34, CD31, and CD45 (BD Biosciences). Fluorescence-activated cell sorting was performed on a BD FACSAria II (BD Biosciences, San Jose, CA, United States), using a 100-μm nozzle. Propidium iodide was used as the live/dead discriminator. Compensation was performed using CD31-PB, CD45-PECy7, and CD43-FITC with antibody capture beads (Thermo Fisher). Acquisition was performed by BD FACSDiva (BD Biosciences). Analysis was performed using FlowJo (version 10). Gating for CD34-FITC positive utilized a full stain minus one control.

### Cell Culture

Aligned nanofibrillar scaffolds (1-cm long) were placed in 24-well ultra-low attachment plate (Corning), and pre-incubated with 1 ml DMEM/10% FBS with 1% penicillin-streptomycin in CO_2_ incubator for 45 min. At the end of the pre-incubation, media was removed, and 1 ml SVF suspension in DMEM/10% FBS with 1% penicillin-streptomycin was added to the wells containing scaffold samples. The scaffolds were incubated with SVF suspension 5% CO_2_ and 37°C for 1 h, then the cell sample in each well was resuspended, and incubated again for 1 h. After the 2-h incubation, cell-seeded scaffold samples were transferred into new wells containing fresh media and incubated in CO_2_ incubator at 37°C overnight. As a control, 1 ml SVF suspension in DMEM/10% FBS with 1% penicillin-streptomycin was incubated at 10^6^ cells/ml/well in 24-well ultra-low attachment plate in CO_2_ incubator at 37°C overnight. After overnight incubation, SVF cells cultured on scaffolds were dissociated using a 1:1 mixture of TrypLE (Fisher Scientific) and collagenase digestion buffer, pelleted, and resuspended in FACS buffer. SVF cells cultured in suspension were pelleted and resuspended in FACS buffer. Both scaffold- and suspension cultured SVF samples were analyzed by flow cytometry as described above.

### VEGF Enzyme Linked Immunosorbent Assay (ELISA)

Following the overnight incubation of control cell suspension and cell-seeded scaffolds, media samples were collected and kept frozen at −80°C. Corresponding cell numbers were determined after counting by hemocytometer for cell suspension samples, and by DNA quantification for cell-seeded scaffold samples. VEGF concentration in media samples was measured by VEGF enzyme linked immunosorbent assay (ELISA) kit (R&D Systems) following manufacturer’s instructions.

### Preparation of Cell-Seeded Scaffolds and Control Cell Suspension for *in vivo* Implantation

Following the overnight incubation, cell-seeded scaffold samples were removed from the media, rinsed in PBS, placed in 1.5-ml Eppendorf tube filled with PBS, and transferred to the surgery room. Control samples were prepared from the SVF samples incubated in 24-well ultra-low attachment plate overnight. Cells were resuspended, centrifuged at 1250 r/min for 5 min at room temperature, and SVF suspension sample was prepared at 10^4^ cells in 50 μl PBS per injection/animal. This cell number was based on pilot studies showed that 1-cm BioBridge scaffold could hold up to 10^4^ cells. Quantification of scaffold-attached cells was performed by measuring DNA content in cell lysates using PicoGreen assay (Fisher Scientific), and calculation based on calibration curves obtained from series of cell samples with known cell numbers counted by hemocytometer.

### Immunofluorescence Staining of SVF-Seeded Scaffolds

Cell-seeded scaffolds were fixed with 4% paraformaldehyde and permeabilized with 0.5% Triton-X-100, then blocked with 1% bovine serum albumin. Scaffolds were incubated with up to two of the following agents to determine the cellular makeup of the SVF. A 1:100 dilution of anti-human CD31 antibody (DAKO, M082301-2), 1:100 dilution anti-CD105 (endoglin) antibody (Santa Cruz Biotechnology, sc-18838), 1:200 dilution anti-human CD34 antibody (Novus Biologicals, NBP2-44568), and 1:100 dilution Alexa Fluor 488 Phalloidin (ThermoFisher, A12379) for F-actin visualization. Samples were washed in PBS and incubated with secondary antibodies conjugated to Alexa Fluor 488 or Alexa Fluor 594 (ThermoFisher). Scaffolds were washed in PBS and counterstained with Hoechst 33342 nuclear dye (Invitrogen, H3570). Scaffolds were then imaged with a confocal microscope (LSM710, Zeiss).

### Hindlimb Ischemia and Blood Perfusion Assessment

Hindlimb ischemia was induced in 8–10 week old male NOD SCID mice (Jackson Laboratory) by unilaterally excising a portion of the femoral artery (Niiyama et al., 2009). The animals then received one of the following treatments: 10^4^ SVF cells pre-seeded onto a 1-cm-long scaffold and delivered to the site of the ligated artery, or 1 × 10^4^ SVF cell delivered to the adductor muscle of the ischemic leg by local intramuscular injection. Acellular scaffold and a PBS (50 μl) injection were included as reference control groups. Limb perfusion was measured up to 14 days post-surgery by using laser Doppler spectroscopy (PIM3, Perimed). Each animal was placed on a warming pad until they reached a core temperature of 37.5°C before imaging (Niiyama et al., 2009). Results were assessed by taking perfusion value of the ischemic foot, relative to that of the non-ischemic foot to obtain a mean perfusion ratio (Huang et al., 2010; Rufaihah et al., 2011; Nakayama et al., 2015; Hou et al., 2018). All animal studies were approved by the Stanford University Administrative Panel on Laboratory Animal Care.

### Immunofluorescence Assessment of SVF Cell Retention

After 14 days, the gastrocnemius tissues were harvested, snap frozen in OCT, and cryosectioned in 10-μm thick sections. To visualize the persistence of human SVF cells, tissue sections were immunofluorescently stained with antibodies directed against human-specific nuclear matrix antigen (Millipore), human-specific CD34 (Novus Biologicals), and human-specific CD31 (Dako). After overnight incubation, the cells were washed in PBS and then incubated with secondary antibodies conjugated to Alexa Fluor 594 or Alexa Fluor 488. The immunofluorescently stained slides were imaged using an epifluorescence microscope (Observer Z1, Zeiss).

### Statistics

Data are shown as mean ± standard deviation. Statistical analysis of SVF in suspension, in comparison to on aligned nanofibrillar scaffolds, was performed using the Wilcoxon non-parametric paired *t*-test. For *in vivo* studies, mean blood perfusion recovery among treatment groups was statistically analyzed using a one-way analysis of variance (ANOVA) with Bonferroni post-test. Statistical significance was accepted at *P* < 0.05.

## Results

### Characterization of Freshly Isolated Bulk SVF by Flow Cytometry

**FIGURE 1:**
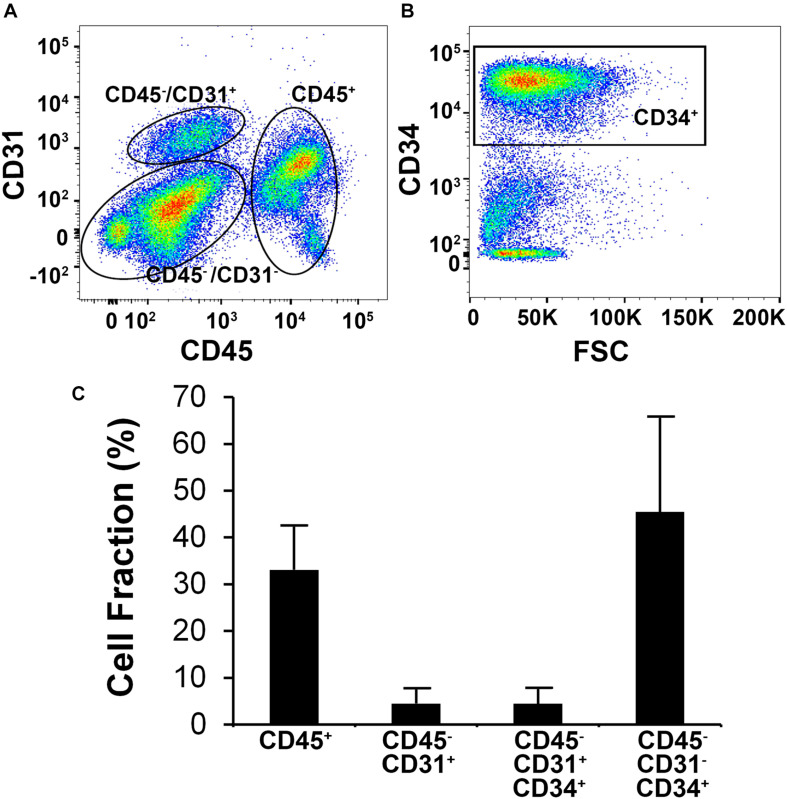
Flow cytometric analysis of human stromal vascular fraction (SVF) cells. **(A)** Representative flow cytometric plot depicts subpopulations of cells based on the expression of CD31 and CD45. **(B)** Flow cytometric analysis of the CD31^–^/CD45^–^ subpopulation showing that the majority of the cells are CD34^+^. **(C)** Mean cell fraction data among six independent donor SVF extractions.

Human SVF from six independent donors were analyzed by flow cytometry to characterize the phenotype of bulk SVF immediately after isolation (Figure 1). Using antibodies targeting phenotypic markers of vascular (CD31 and CD34) and hematopoietic (CD45) lineages, the SVF was found to be heterogeneous and having subpopulations of CD45^+^, CD45^–^/CD31^+^, and CD45^–^/CD31^–^/CD34^+^cells (Figure 1A). The mean distribution of these subpopulations among six donors was 33.1 ± 9.6% for CD45^+^, 4.5 ± 3.1% for CD45^–^/CD31^+^, and 45.4 ± 20.0% for CD45^–^/CD31^–^/CD34^+^ (Figures 1B,C), the latter subpopulation representing the largest segment of SVF. Among CD45^–^/CD31^+^ subset, 85.4 ± 33.7% of the cells co-expressed CD34^+^.

### Characterization of SVF Cellular Attachment on Aligned Nanofibrillar Scaffolds

**FIGURE 2:**
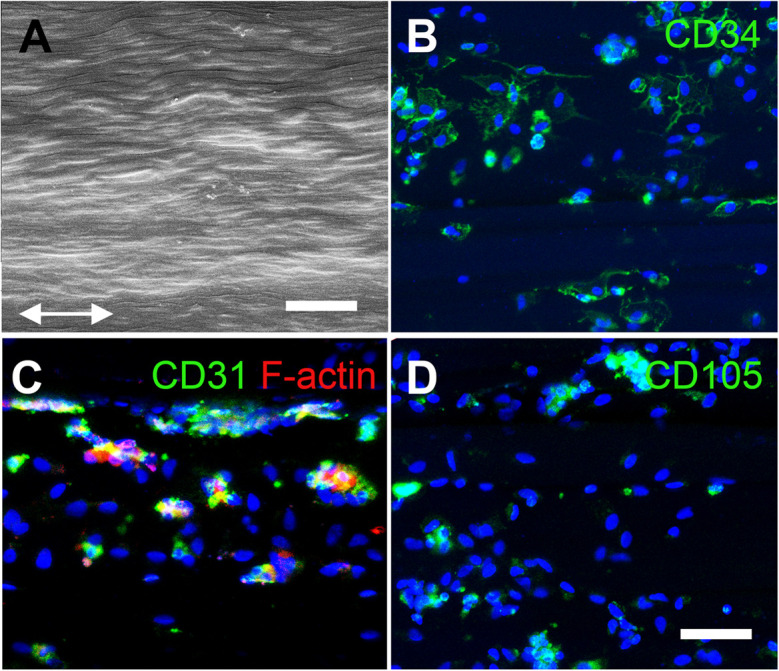
Characterization of human SVF cells on aligned nanofibrillar collagen scaffolds. **(A)** Scanning electron microscopy image depicts nanofibrils arranged in parallel along the direction of the arrows. Confocal images show CD34 **(B)**, CD31 **(C)**, and CD105 **(D)** protein expression among adherent SVF cells on aligned nanofibrillar scaffolds after 1 day of attachment. Scale bars: 2 μm **(A)**; 50 μm **(B–D)**.

**FIGURE 3:**
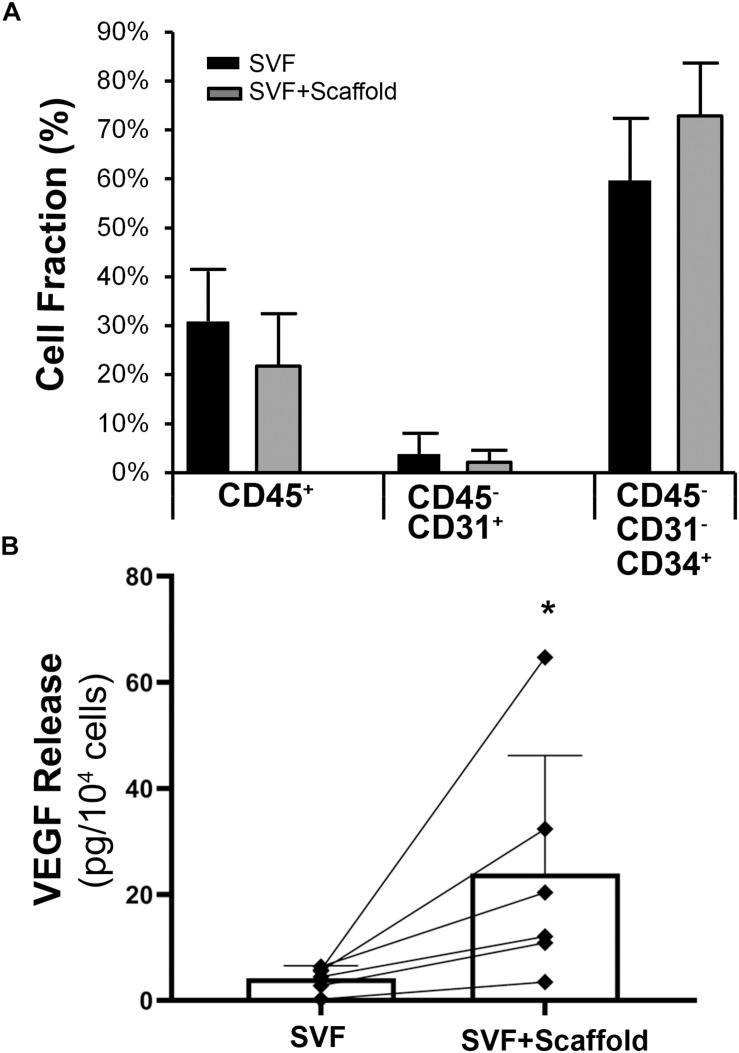
Characterization of adherent SVF cells on aligned nanofibrillar scaffolds. **(A)** Phenotypic markers of adherent cells on aligned nanofibrillar scaffolds, compared to bulk SVF cells in suspension by flow cytometry (*n* = 4). **(B)** Quantification of VEGF protein release in the bulk SVF population, in comparison to the adherent cells seeded on aligned nanofibrillar scaffolds, for six independent donors. * denotes statistically significant relationship, compared to bulk SVF (*p* < 0.05).

Aligned nanofibrillar scaffolds were fabricated using shear-mediated extrusion technology that preferentially induced fibrillogenesis along one principle axis, creating 0.2-mm-wide thread-like scaffolds with longitudinally oriented bundles of nanofibrils. Each individual nanofibril was approximately 200 nm in diameter (Figure 2A), based on SEM imaging. After seeding of the freshly isolated bulk SVF onto the aligned nanofibrillar scaffolds for 1 day, the cell-seeded samples were fixed in 4% paraformaldehyde for immunofluorescence staining of phenotypic markers. As shown by the confocal microscopy images, overnight culture of freshly harvested SVF revealed diverse cellular morphologies, including elongated and adherent cells, loosely adherent cells with rounded morphology, as well as small cellular aggregates (Figures 2B–D). Some of the SVF expressed CD31 and CD34, in agreement with the flow cytometry data (Figures 2B,C). Additionally, CD105, which is expressed by both mesenchymal and endothelial lineages, was also found to be expressed by some cells attached to the aligned nanofibrillar scaffolds (Figure 2D). To quantitatively assess the phenotype of SVF 1 day after attachment to the nanofibrillar scaffold, in comparison to SVF in suspension culture, we performed flow cytometric analysis using the same phenotypic markers. Our results show that the percent population of vascular (CD31 and CD34) and hematopoietic (CD45) phenotypic markers did not significantly change over the course of 1 day of attachment to the scaffold (Figure 3A). Propidium iodide analysis confirmed > 80% viable cells in both treatment groups for all donor cells, which suggested high cell viability.

Although the cells remained phenotypically unchanged after attachment to aligned nanofibrillar scaffolds, the cells became functionally more angiogenic. Among individual SVF donor lines, the average VEGF level from the conditioned media derived from suspension cells was 4.3 ± 1.4 pg per 10^4^ cells (Figure 3B). In stark contrast, the average VEGF secretion from cells seeded on the aligned collagen scaffold was 26.0 ± 23.5 pg per 10^4^ cells, which represented a significant increase by more than a six-fold (*p* < 0.05). For all donor cell lines, there was a consistent increase in VEGF release on scaffold, compared to the bulk SVF suspension population, despite some degree of heterogeneity in the magnitude of VEGF increase among donor cells. These data suggested that aligned nanofibrillar scaffolds could promote angiogenic capacity of SVF.

### Therapeutic Efficacy of Aligned Nanofibrillar Scaffolds Seeded With SVF in a Mouse Model of PAD

**FIGURE 4:**
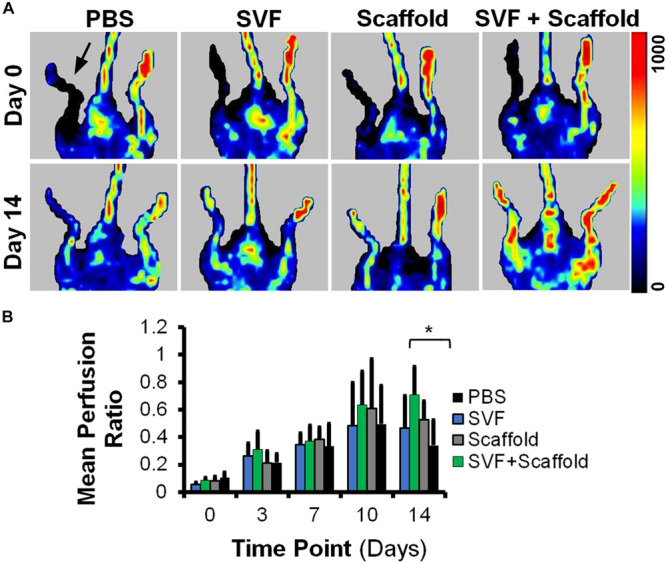
Mean blood perfusion recovery after implantation of aligned nanofibrillar scaffolds seeded with SVF. **(A)** Representative laser Doppler spectroscopy images from day 0 and day 14. **(B)** Quantification of mean perfusion ratio (ischemic/control) over the course of 14 days (*n* = 5–9 per group). ^∗^ denotes statistically significantly higher mean perfusion ratio in the SVF+Scaffold group, relative to the PBS group (*p* < 0.05). Arrow denotes ischemic limb.

**FIGURE 5:**
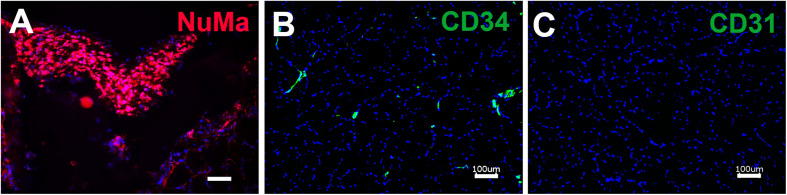
Immunofluorescence staining of retained human SVF adjacent to the site of cell-seeded scaffold transplantation. **(A)** Human SVF are visualized using human specific nuclear matrix antigen (NuMA). Immunofluorescence imaging of human-specific antibodies targeting CD34 **(B)** and CD31 **(C)**. Scale bar: 50 μm **(A)**, 100 μm **(B,C)**.

To determine if the enhanced production of VEGF by SVF on aligned scaffolds could impart a therapeutic benefit, we implanted SVF-seeded scaffolds into immune compromised NOD SCID mice with hindlimb ischemia as an experimental model of PAD. Therapeutic improvement was assessed by non-invasive measurement of blood perfusion recovery by laser Doppler spectroscopy over the course of 14 days after implantation. Quantitative analysis of mean perfusion recovery demonstrated a significant improvement in animals treated with the SVF-seeded scaffolds after 14 days (0.71 ± 0.21), in comparison to animals treated with PBS (0.34 ± 0.18) (Figure 4). In contrast, animals treated with injections of SVF or implantation of the acellular scaffold did not show statistically significant improvement in perfusion recovery, in comparison to animals treated with PBS (Figure 4B). Additionally, SVF could be visualized in some histological tissue sections using human specific nuclear matrix antigen near the site of implantation, suggesting the persistence of these cells (Figure 5A). The SVF cells appeared to retain the expression of CD34 and lacked the expression for CD31, based on staining with human-specific antibodies (Figures 5B,C). The persistence of CD34 and absence of CD31 is consistent with their cellular phenotype prior to transplantation, in which the majority of cells were CD34 with a low incidence of CD31 cells (Figure 3). Together, these results indicated that only SVF-seeded scaffolds promoted significant improvement in blood perfusion recovery, which concurs with the finding of SVF-seeded scaffolds releasing significantly more VEGF (Figure 3B).

## Discussion

The salient results of this study are that SVF-seeded aligned nanofibrillar scaffold significantly improved blood perfusion recovery, compared to a control PBS injection (Figure 4), and that SVF cells attached to aligned nanofibrillar scaffolds produced significantly more VEGF than SVF cells in suspension (Figure 3). This is the first preclinical study that demonstrates that SVF-seeded aligned nanofibrillar scaffold significantly improves blood perfusion, compared to the control PBS injection group. In contrast, treatment with acellular scaffold alone had no therapeutic effect on the level of blood perfusion in the mouse ischemic limb. Likewise, treatment with SVF cellular injection alone did not have a significant benefit over PBS. Therefore, the combination of the SVF cells and aligned nanofibrillar scaffolds could be a promising therapeutic strategy to treat PAD.

Notably, cells attached to the scaffold *in vitro* produced significantly higher levels of VEGF, suggesting that cell attachment to the aligned collagen scaffold may be necessary for inducing VEGF production. This concurs with published literature showing that the gene expression of various pro-angiogenic factors such as VEGF could be regulated using hydrogels of various stiffnesses (Cai et al., 2016). Additionally, in our previous studies, we demonstrated that cellular attachment to aligned nanofibrillar scaffolds increased the gene expression of integrin α_1_ subunit, which has been shown to be upregulated during angiogenesis (Nakayama et al., 2015). These studies highlight a potentially important role of nano-scale extracellular matrix interactions in mediating changes in cellular function. In a recent study, collagen biomaterial has been shown to enhance pro-angiogenic activity of CD34+ cells (McNeill et al., 2019). Together, the published literature supports our finding that mechanical and biophysical properties of biomaterials can modulate cellular secretion of angiogenic growth factors, which can contribute to pro-angiogenic capacity. However, further studies are warranted to elucidate the signaling mechanisms that govern how aligned nanofibrillar collagen scaffolds modulate cellular secretion of growth factors.

Another mechanism that may explain the increased blood perfusion with SVF-seeded scaffold treatment is cell survivability and persistence. In our previous studies, we observed that nanofibrillar scaffolds had increased the survival duration of endothelial cells, and this potentially also applies to other cell types such as SVF cells (Huang et al., 2013b; Nakayama et al., 2015). It is plausible that the nanofibrillar scaffold prolonged the survival of SVF cells, resulting in a longer duration of therapeutic effect (Hoareau et al., 2018). Besides VEGF, other interesting cellular signaling mechanisms may also be at play. Additionally, the small sample size and variability of blood perfusion recovery in this study may have precluded the possibility of observing significant improvements in the other treatment groups.

Previous studies have been conducted with the use of mononuclear cells as a therapy for CLI. Many of such preclinical and clinical studies used a heterogeneous population of mononuclear cells which showed mixed results in resolving limb pain, ulcers, and amputation rates in particular (Matoba et al., 2008; Teraa et al., 2015). Autologous stem cell therapy for PAD using mononuclear cells or MSC derived from bone marrow or peripheral blood improved the ulcer healing rate and reduced amputation rate, but no significant improvement in major limb salvage was reported (Gao et al., 2018). At least one clinical trial with the use of adipose-derived stem cells (ASC) to treat CLI is under way (Clinicaltrials.gov Identifier NCT03968198). ASC are multipotent cells that can differentiate in multiple cell types including endothelial cells (Cao et al., 2005). In addition to the multipotency of ASC, their benefits for vascular regeneration include paracrine secretion of cytokines and growth factors that may stimulate angiogenesis (Park et al., 2008). Pro-angiogenic potential of MSC is generally believed to be mediated by the secretion of multiple paracrine factors, and this property is largely similar between ASC (Park et al., 2008) and bone marrow derived MSC (Kwon et al., 2014).

The advantages of ASC over other stem cells include relative abundance of subcutaneous adipose tissue in many patients, ease of access without significant donor site morbidity (Tobita et al., 2011; Mizuno, 2013), and two orders of magnitude higher yield of cells per g tissue, compared to bone marrow (Mizuno, 2013; Ong and Sugii, 2013). However, MSC from both adipose tissue and bone marrow showed donor variability (Mohamed-Ahmed et al., 2018). Accordingly, the use of autologous ASCs showed a high variation in clinical outcome, due to differences in donor age, gender, and weight, and the anatomic harvest location and depth (Aksu et al., 2008; Baglioni et al., 2012). Although this current study is not powered to evaluate donor-specific outcomes, it is plausible that inherent differences in the quality of donor SVF may lead to different *in vivo* angiogenic outcomes.

Freshly isolated SVF includes an abundant population of ASC, which can be identified as CD34^+^/CD31^–^/CD45^–^ cells (Tevlin et al., 2016; Brett et al., 2017a), and are mostly of pericytic or mesenchymal phenotype (Traktuev et al., 2008). Non-hematopoietic SVF cells also have a small numbers of CD31^+^/CD34^+^ and CD31^+^/CD34^–^ cells, which are generally identified as endothelial progenitor and mature cells, respectively (Zimmerlin et al., 2013; Polancec et al., 2019), although the latter were also referred as co-expressing CD34^+^ (Tallone et al., 2011).

Preclinical data showed that CD34^+^ cells represent a main subset of stem/progenitor cells in peripheral blood mononuclear cell transplants that potentiate neovascularization in the ischemic area (Schatteman et al., 2000), and improve blood perfusion (Li et al., 2010) and wound healing (Tanaka et al., 2018). In the present study, CD45^–^/CD31^–^/CD34^+^ comprised the majority of the SVF cells. To determine the contribution of CD34^+^ SVF cells on revascularization in the setting of limb ischemia model, mechanistic studies in which CD34^+^ cells are depleted can be performed in future studies. Nevertheless, clinical studies of mononuclear stem cell-based therapeutic angiogenesis employed to treat no-option CLI demonstrated that the number of transplanted CD34^+^ cells was an independent predictor of positive outcome (Pan et al., 2019). In addition, in a study of donor variability of MSC proangiogenic efficacy, Kim et al. (2019) found that a subset of paracrine factors including VEGF serve as efficient biomarkers for predicting vascular regenerative efficacy of stromal/stem cells. Together, these studies together suggest a promising therapeutic benefit of CD34^+^ cell therapy.

The SVF population in this study contained a relatively high fraction of CD34^+^ cells, which are pro-angiogenic cells that are known to produce VEGF (Bautz et al., 2000). We also observed that the cells that attached to our nanofibrillar scaffold had a high amount of CD34^+^ cells compared to cells containing other markers CD31 and CD45. Therefore, it is likely that the CD34^+^ cells within the SVF contribute to the observed pro-angiogenic effects *in vitro* and *in vivo*.

## Conclusion

This is the first study to evaluate an SVF-seeded aligned nanofibrillar scaffold as a potential therapy for PAD. *In vitro* the SVF seeded onto aligned scaffolds produced significantly higher levels of VEGF, compared to cells in suspension. Furthermore, *in vivo* the SVF-seeded scaffolds significantly increased blood perfusion after 14 days in a murine hindlimb ischemia model. SVF is a promising candidate for regenerative medicine due to its availability and as a source of pro-angiogenic cells. Our results suggest that aligned nanofibrillar scaffolds play an important role in enhancing the angiogenic potential of therapeutic cells and have important implications in the design of cell-based therapies for treatment of PAD in patients.

## Data Availability Statement

The raw data supporting the conclusions of this article will be made available by the authors, without undue reservation.

## Ethics Statement

The studies involving human participants were reviewed and approved by the Stanford University Institutional Review Board. The patients/participants provided their written informed consent to participate in this study. The animal study was reviewed and approved by Stanford University Administrative Panel on Laboratory Animal Care.

## Author Contributions

CH, TZ, CA, DN, DW, MP, and NH contributed to the conceptual design of the study. CH, TZ, CA, PT, SS, GY, and MB collected and analyzed the data. CH, TZ, CA, DN, DW, MP, and NH interpreted the data. CH, TZ, CA, MP, and NH wrote the manuscript, with input from all authors.

## Conflict of Interest

MP is the Chief Scientific Officer of Fibralign Corporation. SS and TZ are the employees of Fibralign Corporation.

The remaining authors declare that the research was conducted in the absence of any commercial or financial relationships that could be construed as a potential conflict of interest.
